# Tau-mediated axonal degeneration is prevented by activation of the Wld^S^ pathway

**DOI:** 10.1093/braincomms/fcad052

**Published:** 2023-03-09

**Authors:** Katy Stubbs, Ben Batchelor, Lovesha Sivanantharajah, Megan Sealey, Miguel Ramirez-Moreno, Eva Ruiz, Brad Richardson, Victor H Perry, Tracey A Newman, Amritpal Mudher

**Affiliations:** School of Biological Sciences, Faculty of Environment and Life Sciences, University of Southampton, Highfield Campus, Southampton, SO17 1BJ, UK; School of Biological Sciences, Faculty of Environment and Life Sciences, University of Southampton, Highfield Campus, Southampton, SO17 1BJ, UK; School of Biological Sciences, Bangor University, Bangor LL57 2UW, UK; School of Biological Sciences, Faculty of Environment and Life Sciences, University of Southampton, Highfield Campus, Southampton, SO17 1BJ, UK; School of Biological Sciences, Faculty of Environment and Life Sciences, University of Southampton, Highfield Campus, Southampton, SO17 1BJ, UK; School of Biological Sciences, Faculty of Environment and Life Sciences, University of Southampton, Highfield Campus, Southampton, SO17 1BJ, UK; School of Biological Sciences, Faculty of Environment and Life Sciences, University of Southampton, Highfield Campus, Southampton, SO17 1BJ, UK; School of Biological Sciences, Faculty of Environment and Life Sciences, University of Southampton, Highfield Campus, Southampton, SO17 1BJ, UK; Clinical and Experimental Science, Faculty of Medicine, University of Southampton, Highfield Campus, Southampton SO17 1BJ, UK; School of Biological Sciences, Faculty of Environment and Life Sciences, University of Southampton, Highfield Campus, Southampton, SO17 1BJ, UK

**Keywords:** tauopathy, axonal degeneration, Wallerian degeneration, Wld^S^

## Abstract

Tauopathy is characterized by neuronal dysfunction and degeneration occurring as a result of changes to the microtubule-associated protein tau. The neuronal changes evident in tauopathy bear striking morphological resemblance to those reported in models of Wallerian degeneration. The mechanisms underpinning Wallerian degeneration are not fully understood although it can be delayed by the expression of the slow Wallerian degeneration (Wld^S^) protein, which has also been demonstrated to delay axonal degeneration in some models of neurodegenerative disease. Given the morphological similarities between tauopathy and Wallerian degeneration, this study investigated whether tau-mediated phenotypes can be modulated by co-expression of Wld^S^. In a *Drosophila* model of tauopathy in which expression of human 0N3R tau protein leads to progressive age-dependent phenotypes, Wld^S^ was expressed with and without activation of the downstream pathway. The olfactory receptor neuron circuit *OR47b* was used for these studies in adults, and the larval motor neuron system was employed in larvae. Tau phenotypes studied included neurodegeneration, axonal transport, synaptic deficits and locomotor behaviour. Impact on total tau was ascertained by assessing total, phosphorylated and misfolded tau levels by immunohistochemistry. Activation of the pathway downstream of Wld^S^ completely suppressed tau-mediated degeneration. This protective effect was evident even if the pathway downstream of Wld^S^ was activated several weeks after tau-mediated degeneration had become established. Though total tau levels were not altered, the protected neurons displayed significantly reduced MC1 immunoreactivity suggestive of clearance of misfolded tau, as well as a trend for a decline in tau species phosphorylated at the AT8 and PHF1 epitopes. In contrast, Wld^S^ expression without activation of the downstream protective pathway did not rescue tau-mediated degeneration in adults or improve tau-mediated neuronal dysfunction including deficits in axonal transport, synaptic alterations and locomotor behaviour in tau-expressing larvae. This collectively implies that the pathway mediating the protective effect of Wld^S^ intersects with the mechanism(s) of degeneration initiated by tau and can effectively halt tau-mediated degeneration at both early and late stages. Understanding the mechanisms underpinning this protection could identify much-needed disease-modifying targets for tauopathies.

## Teaser

This study investigated whether expression of the axon-protective slow Wallerian protein (Wld^S^) protects against axonal degeneration following expression of pathological human tau protein in a *Drosophila* model of tauopathy. Whereas simple co-expression of Wld^S^ did not reduce axonal degeneration, activation of the pathway downstream of Wld^S^ abolished tau-mediated degeneration in Tau;Wld^S^ bigenics.

## Graphical Abstract

**Graphical Abstract:**
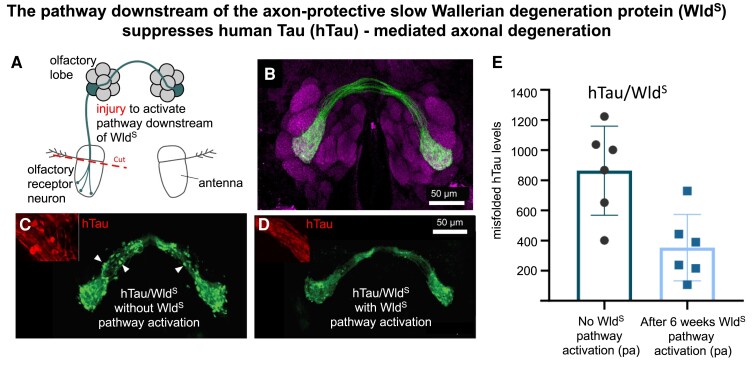

## Introduction

Tau pathology is observed in numerous neurodegenerative diseases, including Alzheimer’s disease, Parkinson’s disease, motor neuron disease and a variety of other tauopathies such as frontotemporal dementia, Pick’s disease, progressive supra-nuclear palsy and others. The axon is susceptible to tau pathology in these neurodegenerative diseases, with evidence of white matter changes indicative of axonal degeneration in tauopathies such as Alzheimer’s disease.^1‐3^ Studies in animal models have demonstrated that axonal dysfunction in tauopathy is typified by disrupted axonal transport,^4‐6^ due to tau hyperphosphorylation resulting in reduced cytoskeletal integrity.^7^ Additionally, axonal swellings and loss of white matter, hallmarks of axonal degeneration have been observed in P301L-tau mice, a model of familial frontotemporal dementia.^8‐10^

Wallerian degeneration describes the sequential degeneration of axons following axonal injury that begins with the breakdown of the cytoskeleton and ends with the fragmentation and loss of the separated distal axon.^11^ Wallerian degeneration and axonal degeneration in neurodegenerative disease share similarities including cytoskeletal breakdown,^7,12^ disrupted axonal transport,^13,14^ alterations to mitochondrial morphology^15,16^ and, in the central nervous system (CNS), axonal swellings.^12,17^ These similarities suggest that the mechanisms overlap and the term Wallerian-like is used to describe degeneration that is not due to an acute injury.

An understanding of the molecular events that drive axonal degeneration began with the discovery of the slow Wallerian degeneration (Wld^S^)-protective mutation, which robustly delays Wallerian degeneration,^18^ after an acute injury (reviewed in Conforti *et al.*,^19^ Ding and Hammarlund,^20^ Llobet Rosell and Neukomm^21^ and Coleman and Hoke^22^). Wld^S^ is a chimeric fusion protein resulting from a spontaneous mutation that was identified by one of our team in a unique mouse strain that displayed delayed axonal degeneration following injury.^18^ It is a fusion of the full length of nicotinamide mononucleotide adenylyl transferase 1 (Nmnat1) and the *N*-terminal portion of the ubiquitination factor E4b (Ube4b) linked together by an 18-amino acid sequence from the *Nmnat1* 5′ untranslated region. Wld^S^ was confirmed as the genetic source of the protective phenotype in the unique mouse strain from which it was identified by the generation of the Wld^S^ transgenic mouse, which recapitulated this delayed degeneration phenotype.^23^ Since then, Wld^S^ has been the subject of several studies aimed at understanding the molecular mechanism(s) underpinning its gain of function neuroprotective effect and the insights that this provides about the processes governing axonal degeneration. The demonstration that Wld^S^ delayed axonal degeneration after injury in *Drosophila*^24,25^ proved that the axon death pathway downstream of Wld^S^ is conserved from flies to mammals and allowed the genetic tractability of *Drosophila* to be harnessed to identify key players in this pathway. Such studies have revealed several proteins that play essential roles in maintaining axonal integrity (e.g. NMNAT^26^) or driving axonal destruction [e.g. sterile-alpha and TIR motif containing 1 (SARM),^27,28^ Pebbled,^29^ Highwire^30^ and Axundead^31^]. A prominent role of mitochondrial dysfunction^32^ and NAD^+^ depletion^33^ in triggering this axon death pathway has also been uncovered.

As Wallerian-like degeneration is evident in a variety of neurodegenerative conditions, Wld^S^ has been studied in experimental models of these conditions to explore whether it can protect against this chronic axonal degeneration as it does in acute injury-induced axonal degeneration. This work has demonstrated delayed degeneration following Wld^S^ expression in models of disease including multiple sclerosis,^34^ Parkinson’s disease,^35,36^ Charcot–Marie–Tooth disease type 1A^37^ and 1B^38^ and toxic neuropathy.^39^

Considering that the axon is a site of tau-mediated dysfunction and degeneration, the aim of the present study was to investigate whether the axonal protection mediated by Wld^S^ was able to rescue tau-mediated axonal dysfunction and degeneration. As discussed above, *Drosophila melanogaster* has been used in the study of Wallerian degeneration and Wld^S^,^26,27,30,31,40,41^ and *Drosophila* models of tauopathy are similarly well-established.^5,42,43^ Furthermore, several studies are beginning to implicate components of Wld^S^ (such as nicotinamide mononucleotide adenylyl transferase—NMNAT) in tau-mediated aggregation and degeneration in both rodent and *Drosophila* models.^44‐46^ To investigate whether Wallerian-like degeneration in tauopathy is Wld^S^ sensitive, we studied the structural and functional effects of co-expression of human tau (hTau^0N3R^) and Wld^S^ in *Drosophila*.

Our findings demonstrate that simple co-expression of Wld^S^ does not confer protection against hTau-mediated dysfunction or degeneration. However, in stark contrast, activation of the pathway downstream of Wld^S^ results in profound protection, both preventing and arresting degeneration even in neurons already affected by tau-induced pathology.

## Materials and methods

### Fly stocks

*Drosophila* were raised and maintained on standard Bloomington media at 23°C with a 12-/12-h light/dark cycle. UAS-hTau^0N3R^, D42-GAL4 and Oregon-R flies were obtained from the Bloomington Drosophila Stock Centre (Indiana, IN, USA). The UAS-Wld^S^ transgenic line contains the mouse Wld^S^ Ube4b/Nmnat chimeric gene coding sequence described by Mack *et al.*,^23^ and it was created and characterized by the Luo lab as described in Hoopfer *et al*.^25^ This line and the Or47b-GAL4/Cyo;UAS-mCD8::GFP lines were obtained from Professor Liqun Luo (Stanford University, CA, USA). The D42-GAL4.UAS-NPY::GFP line was generated previously,^5^ with UAS-NPY::GFP provided by Dr. Ian Robinson (Plymouth University, UK). A homozygous hTau^0N3R^;Wld^S^ line was generated for the current study by crossing UAS-hTau^0N3R^ with UAS-Wld^S^ lines. Males were used for all studies in adults.

### Axonal transport analysis

Transgenes were expressed using D42-GAL4.UAS-NPY::GFP, and third-instar wandering larvae were selected for analysis. Larvae were anaesthetized using diethyl ether vapour (Thermo Fisher Scientific) and mounted in 1% agarose (Sigma-Aldrich) on glass slides, with the ventral surface facing the coverslip. Peripheral nerves were imaged using an Axioplan2 MOT upright fluorescence microscope (Zeiss) equipped with Micro Max CCD (Princeton Instruments) using MetaMorph acquisition software (Molecular Devices). Images were thresholded and the area covered by aggregates measured using MetaMorph software.

### Larval NMJ analysis

Transgenes were expressed using D42-GAL4, and third-instar wandering larvae were dissected, with internal organs removed and the skin pinned out and fixed in 4% formaldehyde (Sigma-Aldrich) for 90 min at room temperature. Larval skins were then washed in 0.1% Triton X (Sigma-Aldrich) in phosphate-buffered saline (PBS-Tx; Thermo Fisher Scientific) prior to blocking in 5% goat serum, 3% horse serum and 2% bovine serum albumin (BSA; Sigma-Aldrich) in 0.1% PBS-Tx. Skins were incubated with goat anti-horseradish peroxidase (1:1000; ICN/Cappel), conjugated to fluorescein isothiocyanate. Skins were washed in 0.1% PBS-Tx and put through an ascending glycerol series (50%, 70%, 90% and 100%) before being mounted in Vectashield (Vector Laboratories) and imaged. Neuromuscular junctions (NMJs) on Muscle 4 from Segments A3–5 were imaged using a Leica SP2 scanning confocal microscope using the 488 argon laser. Maximum projections of Z stacks were generated for morphometric analysis; bouton size and interbouton axon width were measured using ImageJ with the assessor blinded to the sample number.

### Larval locomotion

Larval behaviour was assessed as previously described.^47^ In brief, D42-GAL4-driven third-instar wandering larvae were each placed in the centre of 0.3% alcian blue (Sigma-Aldrich), 1% agarose (Sigma-Aldrich) plates and videos of larval behaviour recorded. Videos of larval locomotion were analysed using EthoVision 3.0 (Noldus) tracking software.

### Immunohistochemical analysis of axonal degeneration

Adults were collected 0–2 days after eclosion from Or47b-GAL4, UAS-mCD8::GFP-driven crosses and aged to the relevant time point. Flies were anaesthetized with CO_2_, heads were ligated, and the brains were dissected and placed in 4% formaldehyde and fixed at room temperature for 45 min. Following fixation, brains were washed in 0.1% PBS-Tx before either mounting in Vectashield or proceeding for staining. Those to be stained were blocked in 5% goat serum, 3% horse serum and 2% BSA in 0.1% PBS-Tx and stained with rabbit anti-human tau antibodies (1:1000; Dako) or mouse anti-phospho tau PHF-1 (1:1000; Thermo Fisher), and anti-mouse MC1 (1 in 200—a kind gift from Prof. Peter Davies, Feinstein Institute for Medical Research, USA) washed and incubated in goat anti-rabbit or anti-mouse Alexa Fluor 563 (1:1000; Invitrogen; Thermo Fisher Scientific). Brains were washed and mounted in Vectashield prior to imaging on an Axioplan2 MOT upright epifluorescence microscope (Zeiss) equipped with a QImaging Retiga 3000 CCD Camera (Photometrics) and images were acquired using MetaMorph software (Molecular Devices). Images were quantified in ImageJ with the assessor blinded to genotype and time point. For axonal swellings, images were thresholded, and the coverage of swellings was measured. All genotypes were always treated in exactly the same way, with the same reagents used for all samples where possible, to eliminate experimental bias.

### Axon injury to activate the pathway downstream of Wld^S^ (referred to as Wld^S^ pa)

The third antennal segment was removed from flies, 1 or 3 weeks after eclosion (wae) from Or47b-GAL4-driven crosses, under CO_2_ anaesthesia using Dumont #5 forceps. This induced an axonal injury in ç, whose cell bodies are located in the third antennal segment. At the relevant time points, brains were dissected as described above. Degeneration was quantified by previously described methods.^24^ Briefly, with the assessor blind to genotype and time point, the presence of the commissural axons was recorded (Y/N), and the percentage of brains of each genotype at each time point with intact axons was calculated. The intensity of GFP signal within glomeruli was measured using ImageJ, and the background intensity was subtracted.

### Statistical analysis

Statistical analysis was conducted using GraphPad Prism, version 6.0 (GraphPad Software, Inc.), using analysis of variance and the Bonferroni correction for the comparison of groups. The Mantel–Cox test was used for survival analysis, with the Bonferroni correction used for the comparison of multiple groups. Values are presented as the mean ± standard error. *P* < 0.05 was considered to indicate a statistically significant difference.

## Results

### Expression of Wld^S^ without ‘activation’ of the downstream pathway is insufficient to protect against hTau^0N3R^-induced phenotypes

Though previous studies of injury models indicate that the presence of Wld^S^ within the axon is crucial for its protection,^48^ the findings from chronic models of disease do not show any consistent or significant Wld^S^-mediated protection despite clear evidence of Wallerian-like degeneration in these models.^49‐52^ One explanation for this lack of rescue could be that the pathway that Wld^S^ is acting in is not activated in these models of chronic degeneration, raising the possibility that the protein may require some form of acute insult to unmask or induce its protective effect. Indeed, in all cases where Wld^S^ has been reported to rescue axonal degeneration, the neurons were exposed to an acute insult or injury as part of the experimental paradigm.^35,39,53,54^

**Figure 1:**
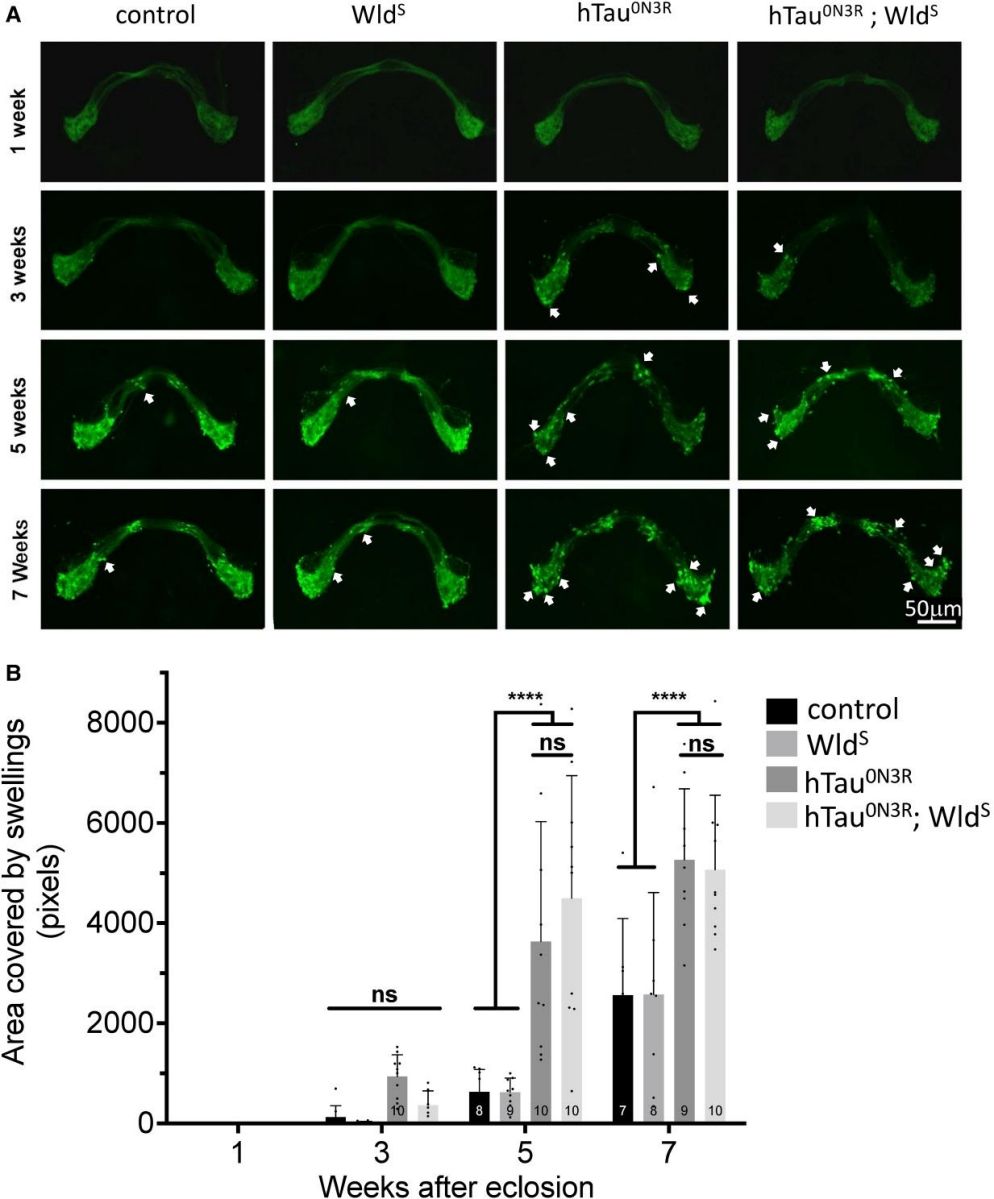
**Simple co-expression of Wld^S^ with hTau^0N3R^ does not delay tau-mediated axonal degeneration.** At 1 week after eclosion, all genotypes display normal olfactory receptor neuron (ORN) morphology as visualized by CD8:GFP immunoreactivity (**A**). At 3 weeks after eclosion, axonal swellings (arrows) are apparent in hTau^0N3R^-expressing ORNs with similar morphology observed in hTau^0N3R^;Wld^S^ ORNs. (**B**) Quantification of swelling coverage indicates that co-expression of Wld^S^ does not delay the onset nor slow the progression of tau-mediated axonal degeneration. Values are presented as the mean ± SD. *n* = 6–11; each data point corresponds to an animal; ****P < 0.0001 (ANOVA with Bonferroni’s multiple comparisons). Driver line for all genotypes was Or47b-GAL4/Cyo; UAS-mCD8::GFP; control was driver crossed with Oregon-R. hTau^0N3R^, 0N3R human tau isoform; n.s., not significant; Wld^S^, slow Wallerian degeneration.

To investigate this, the impact of Wld^S^ expression on hTau^0N3R^-mediated degeneration was examined both in naïve animals, as well as in those where the Wld^S^ pathway was activated by an acute injury paradigm. To study degeneration in the absence of activation of the Wld^S^ pathway, the morphology of naïve olfactory receptor neurons (ORNs) expressing membrane-bound GFP in animals that were also expressing either hTau^0N3R^ alone or hTau^0N3R^ and Wld^S^ (hTau^0N3R^;Wld^S^) was studied at various time points. The ORNs underwent progressive age-related axonal degeneration in all hTau^0N3R^-expressing flies. This was characterized by the appearance of axonal swellings at 2–3 weeks after eclosion, which increased in number and size as the flies aged (arrows Fig. 1A and B hTau^0N3R^ column). Axonal swellings were also evident in controls and Wld^S^ flies, but only at older, 5- to 7-week time points (arrows Fig. 1A control and Wld^S^ columns). Noticeably, the swellings were also apparent in hTau^0N3R^;Wld^S^ flies (where the Wld^S^ pathway had not been activated—Fig. 1A, hTau^0N3R^;Wld^S^ column), and these were similar to those found in the hTau^0N3R^ animals. Quantification confirmed that there was no significant difference in onset, extent or progression of axonal swellings in the hTau^0N3R^;Wld^S^ flies when compared with hTau^0N3R^ alone (arrows Fig. 1A and B).

**Figure 2:**
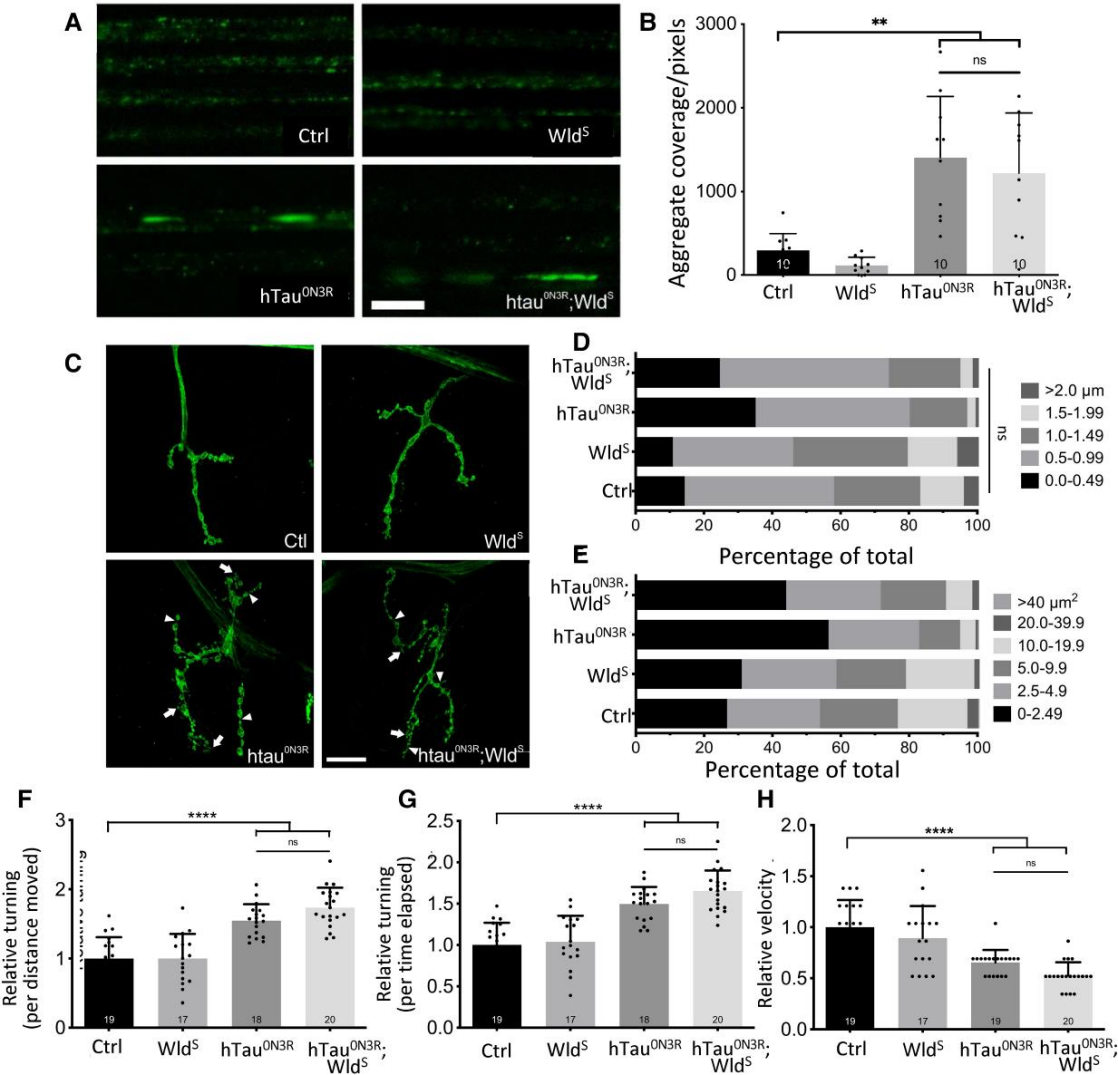
**Simple co-expression of Wld^S^ with hTau^0N3R^ does not improve tau-mediated axonal dysfunction.** (**A**) Expression of hTau^0N3R^ results in the appearance of vesicular aggregates, visualized by neuropeptide Y::GFP fluorescence *in vivo* in anaesthetized larvae, which are also apparent in hTau^0N3R^;Wld^S^ axons (scale bar = 10 µm). (**B**) No significant improvement in aggregate coverage was observed in hTau^0N3R^;Wld^S^ axons compared with hTau^0N3R^ axons. *n* = 10 larvae; each data point corresponds to an animal; *P* > 0.05 (ANOVA with Bonferroni’s multiple comparison). (**C**) hTau^0N3R^ NMJs display aberrant morphology, visualized by CD8::GFP immunostaining, typified by thinning of the axon (arrowheads) and microsatellite boutons (arrows), with this also observed in htau^0N3R^;Wld^S^ NMJs (scale bar = 25 µm). Co-expression of Wld^S^ with hTau^0N3R^ did not rescue thinning of the inter-bouton axon (**D**) or the alterations in bouton size (**E**) *n* = 4 larvae, three to six NMJs/larva *P* > 0.05 (ANOVA with Bonferroni’s multiple correction). Analysis of locomotor behaviour indicated that co-expression of hTau^0N3R^;Wld^S^ did not significantly improve the hTau^0N3R^-mediated alterations in (**F**) meander—relative turning/distance travelled, (**G**) angular velocity—relative turning/time elapsed and (**H**) velocity of larval crawling (*n* > 17; each data point corresponds to an animal). Values are presented as the mean ± SD. **P < 0.01; ****P < 0.0001 (ANOVA with Bonferroni’s multiple correction). Driver line for data presented in **A** and **B** was D42-GAL4.UAS-NPY::GFP; driver line for all other data was D42-GAL4.UAS-mCD8::GFP. Controls were driver lines crossed with Oregon-R. Ctl, control; hTau^0N3R^, 0N3R human tau isoform; NMJs, neuromuscular junctions; Wld^S^, slow Wallerian degeneration.

These data imply that simple co-expression of Wld^S^ does not confer any protection against tau-mediated degeneration in the adult. However, to investigate whether Wld^S^ would protect against tau-mediated dysfunction in larvae, which is highly dependent upon pathogenic effects of human tau in the axon, the impact of Wld^S^ expression on established axonal and synaptic phenotypes was investigated. In previous studies, the expression of hTau^0N3R^ in *Drosophila* motor neurons resulted in disrupted axonal transport, characterized by the appearance of vesicular aggregates.^5^ Using a *Drosophila* line expressing GFP-tagged neuropeptide Y in motor neurons, axonal transport was visualized using microscopy in live intact third-instar larvae. As reported previously, numerous large vesicular aggregates were found in tau-expressing larvae (Fig. 2A). However, these aggregates were also observed in hTau^0N3R^;Wld^S^ larvae. Quantification indicated that the area of the aggregates were not significantly reduced in hTau^0N3R^;Wld^S^ larvae compared with hTau^0N3R^ larvae (Fig. 2B).

HTau^0N3R^ expression is associated with altered synaptic morphology, characterized by thinning of the inter-bouton axons (Fig. 2C) and the appearance of minisatellite boutons (arrowheads in Fig. 2C),^55^ features that were observed in the current study. This phenotype was not improved by co-expression of Wld^S^; no significant difference in the thickness of the inter-bouton axon (Fig. 2D) or the proportions of bouton of each size (Fig. 2E) was seen in the NMJs of either hTau^0N3R^;Wld^S^- or hTau^0N3R^-expressing animals.

Disruption of axonal transport and altered synaptic morphology are associated with alterations in locomotor behaviour.^5^ Using an open-field behavioural assay, the crawling behaviour of third-instar larvae was investigated. When placed in the centre of an arena, control larvae quickly move towards the edge of the arena, following a straight path. In contrast, tau-expressing larvae move more slowly and take a confused and twisting path, demonstrated by an increase in meander (turning per distance moved; Fig. 2F) and angular velocity (turning per time elapsed; Fig. 2G) and a reduction in overall velocity (Fig. 2H). However, the co-expression of Wld^S^ with hTau^0N3R^ did not improve locomotor behaviour, with no significant difference between hTau^0N3R^;Wld^S^ and hTau^0N3R^-expressing larvae in any of these parameters (Fig. 2F–H).

These data show that simply by expressing Wld^S^ with hTau^0N3R^, its protective effect on hTau^0N3R^-mediated dysfunction (in larvae) or degeneration (in adult flies) is not evident.

### Activation of the pathway downstream of Wld^S^ protects against hTau^0N3R^-induced degeneration

**Figure 3:**
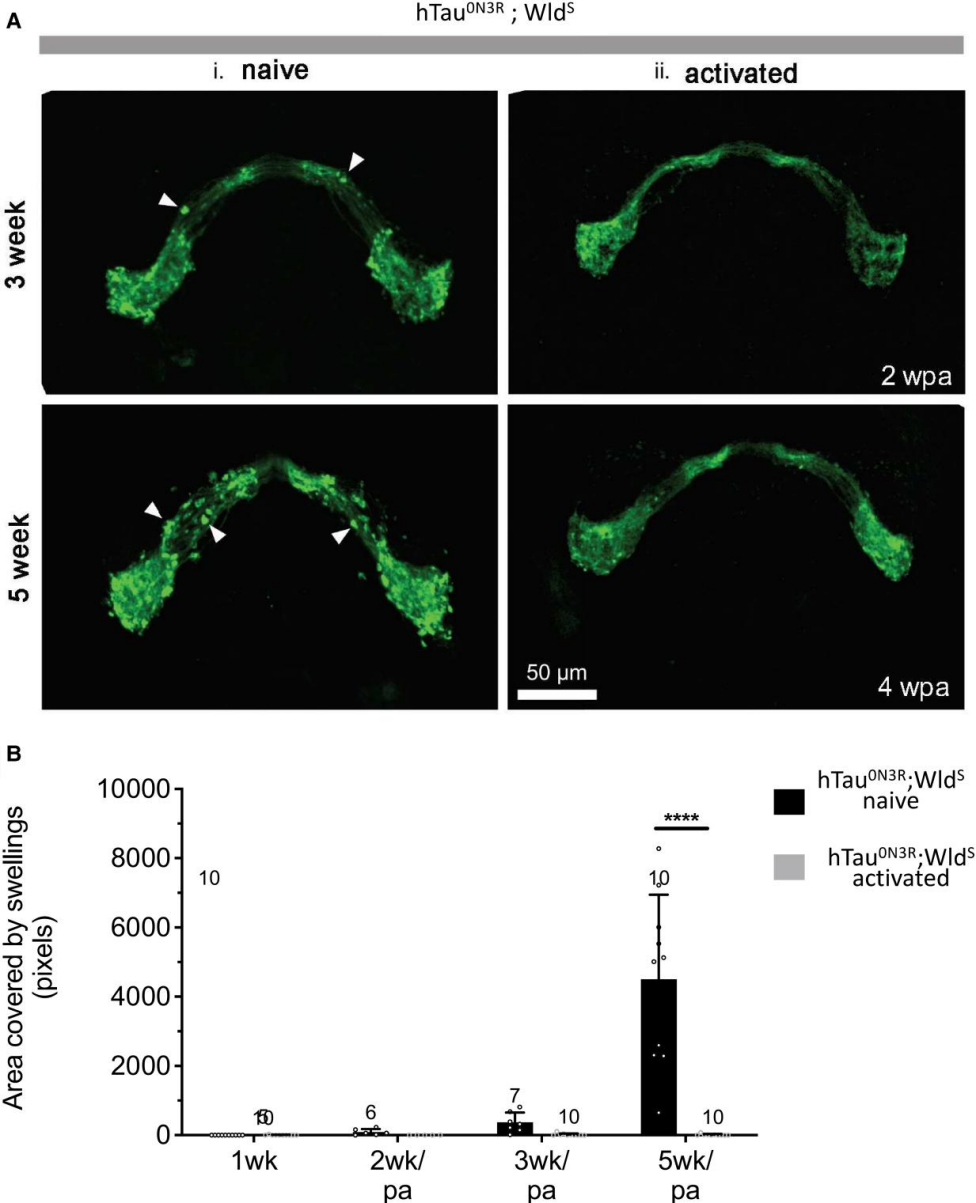
**Tau-mediated axonal swellings are not evident in hTau^0N3R^;Wld^S^ axons in which Wld^S^ pathway is activated.** (**A**) Anti-GFP staining to identify axonal swellings (arrowheads) in CD8::GFP-expressing naïve htau^0N3R^;Wld^S^ axons where Wld^S^ pathway has not been activated. These swellings do not appear in cd8::GFP-expressing hTau^0N3R^;Wld^S^ axons where Wld^S^ has been activated, at any time point pathway activation (pa). (**B**) Quantification of coverage of axonal swellings. Values are presented as the mean ± SD. *n* = 6–10; each data point corresponds to an animal. *P* < 0.0001 (ANOVA with Bonferroni’s multiple comparisons). hTau^0N3R^, 0N3R human tau isoform; Wld^S^, slow Wallerian degeneration.

To explore whether injury-induced activation was required for the Wld^S^ pathway’s protective effect against tau-mediated degeneration, the axons of the hTau^0N3R^;Wld^S^ ORNs were injured (axotomized) by removal of the third antennal segments as previously described.^24,25^ Adult brains were analysed after eclosion at hourly (h) or weekly (w) time points post-axotomy induced Wld^S^ pathway activation (referred to as ‘pa’ from here on). This revealed that control and tau-expressing axons had degenerated at 2 weeks after eclosion/1-week pa; in contrast, both Wld^S^ and hTau^0N3R^;Wld^S^-expressing axons were intact at this time point (Supplementary Fig. 1). This confirms that the axotomy paradigm activated the pathway downstream of Wld^S^ in all axons that express Wld^S^ even if other proteins like tau were being co-expressed. To ascertain the extent to which activation of the Wld^S^ pathway protected against tau-mediated degeneration, the axonal swellings that were the prominent feature of tau-expressing axons (described in Fig. 1) were quantified. As was noted in Fig. 1, conspicuous axonal swellings were present in naïve hTau^0N3R^;Wld^S^ axons that increased with time, suggestive of progressive tau-mediated degeneration (arrowheads Fig. 3A, i). In contrast, these were not found in hTau animals of the same genotype after activation of the Wld^S^ pathway (Fig. 3A, ii). The progressive accumulation of these axonal swellings is evident in hTau-expressing animals within 2 weeks after eclosion and trebles by Week 5. In contrast, swellings were not seen at any time point in hTau^0N3R^;Wld^S^-expressing animals where the activation of the Wld^S^ pathway had been elicited (Fig. 3B). This illustrates that once activated, the Wld^S^ pathway protects against the initiation and development of tau-mediated axonal degeneration.

**Figure 4:**
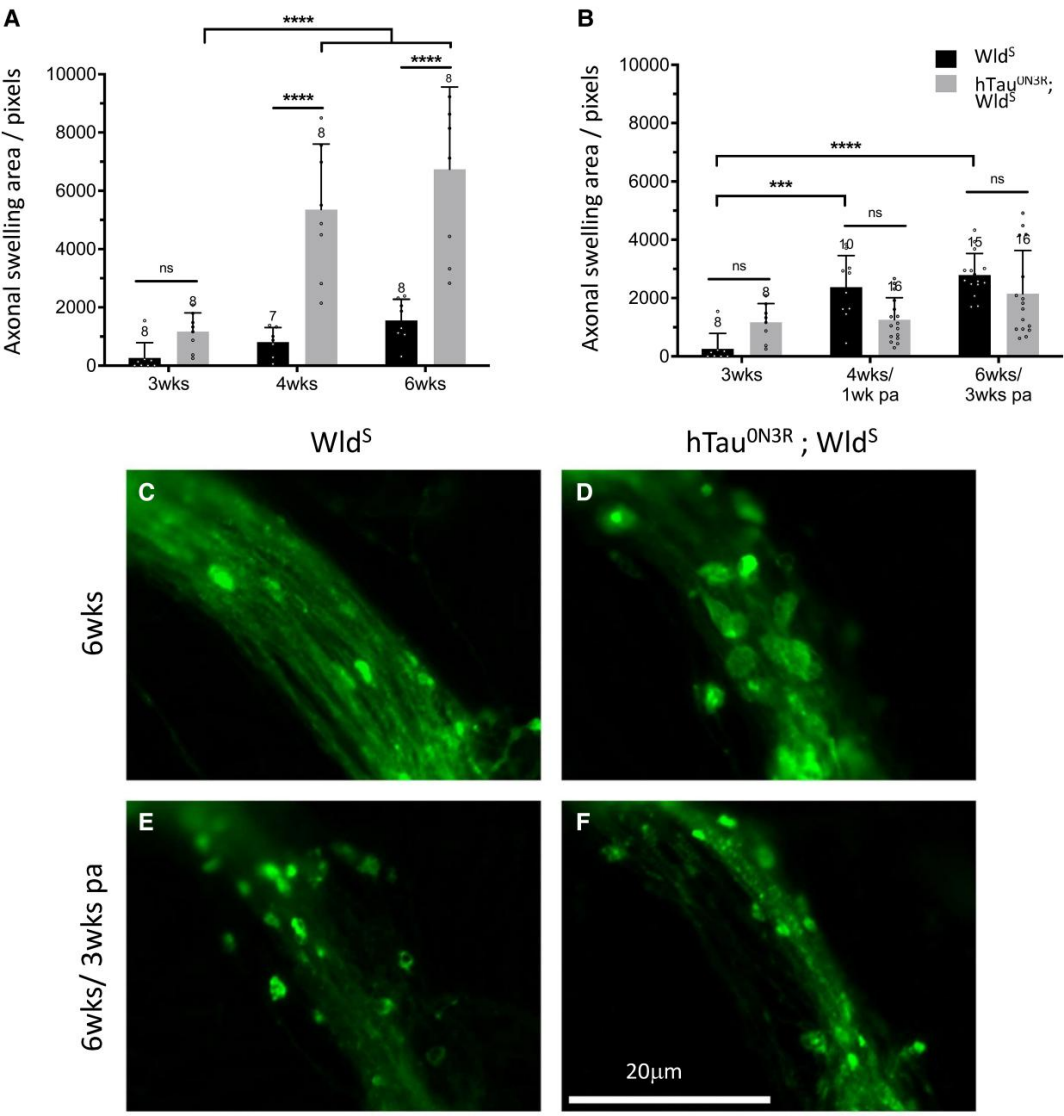
**Tau-mediated axonal swellings are halted from progressing upon activation of the Wld^S^ pathway.** (**A**) Quantification of CD8::GFP immunostaining to visualize axonal swellings reveals that in naïve hTau^0N3R^;Wld^S^ axons, where Wld^S^ pathway has not been activated, the level of swellings increases significantly at 4 and 6 weeks (*n* = 7–8; each data point corresponds to an animal). (**B**) In hTau^0N3R^;Wld^S^ axons where Wld^S^ has been activated, the area covered by axonal swellings does not increase significantly over time post-Wld^S^ pathway activation (pa) (*n* = 8–16; each data point corresponds to an animal). (**C–F**) Representative images from two time points. Values are presented as the mean ± SD. ***P < 0.001, ****P < 0.0001 (ANOVA with Bonferroni’s multiple comparisons). hTau^0N3R^, 0N3R human tau isoform; Wld^S^, slow Wallerian degeneration.

To study a more disease-relevant situation, we investigated whether activation of the Wld^S^ pathway protected against already established tau-mediated axonal degeneration. The Wld^S^ pathway was activated at 3 weeks after eclosion, a time at which axonal swellings are already established in hTau^0N3R^-expressing axons. In hTau^0N3R^;Wld^S^ axons where the Wld^S^ pathway was not activated, a progressive increase in axonal swellings is evident with time, such that axonal swellings at 4 weeks after eclosion are 4-fold greater than those seen at 3 weeks after eclosion, with this increasing further by 6 weeks after eclosion (*P* < 0.001; Fig. 4A). At these later time points, the swellings in the naïve hTau^0N3R^;Wld^S^ axons are significantly greater than those seen in the naïve Wld^S^ axons that serve as the controls. In contrast, activation of the Wld^S^ pathway halts this further development of axonal swelling (Fig. 4B). ORNs in flies expressing hTau;Wld^S^ showed the anticipated accumulation of axonal swellings at 3 weeks after eclosion, prior to Wld^S^ pathway activation, but any further accumulation was halted once the Wld^S^ pathway was activated (Fig. 4B) with no progression in pathology seen after this time. Once the pathway downstream of Wld^S^ was activated, the axonal swellings in hTau^0N3R^;Wld^S^ animals (Fig. 4B, grey bars) were dramatically reduced compared to those evident in similarly aged naïve hTau^0N3R^;Wld^S^ animals (Fig. 4A, grey bars). Indeed after activation of the pathway downstream of Wld^S^, the axonal swelling in the hTau^0N3R^;Wld^S^ animals were not significantly different to those seen in controls at any time point (Fig. 4B) (representative images shown in Fig. 4C–F). This indicates that in addition to preventing the emergence of tau-mediated axonal degeneration (as shown in Fig. 3), activation of the Wld^S^ pathway can also halt the progression of tau-mediated axonal degeneration once it has begun.

The results indicate that activation of the pathway downstream of Wld^S^ potently suppresses hTau-mediated degeneration. This supports our hypothesis that the lack of any protection through co-expression of Wld^S^ in the chronic models of degeneration that have been studied^49‐52^ is because the pathway that Wld^S^ acts in is not normally activated in otherwise naïve axons. Acute injury or other acute insult to an axon activates it unmasking its protective effect.

### Impact of the activation of the Wld^S^ pathway on hTau^0N3R^

**Figure 5:**
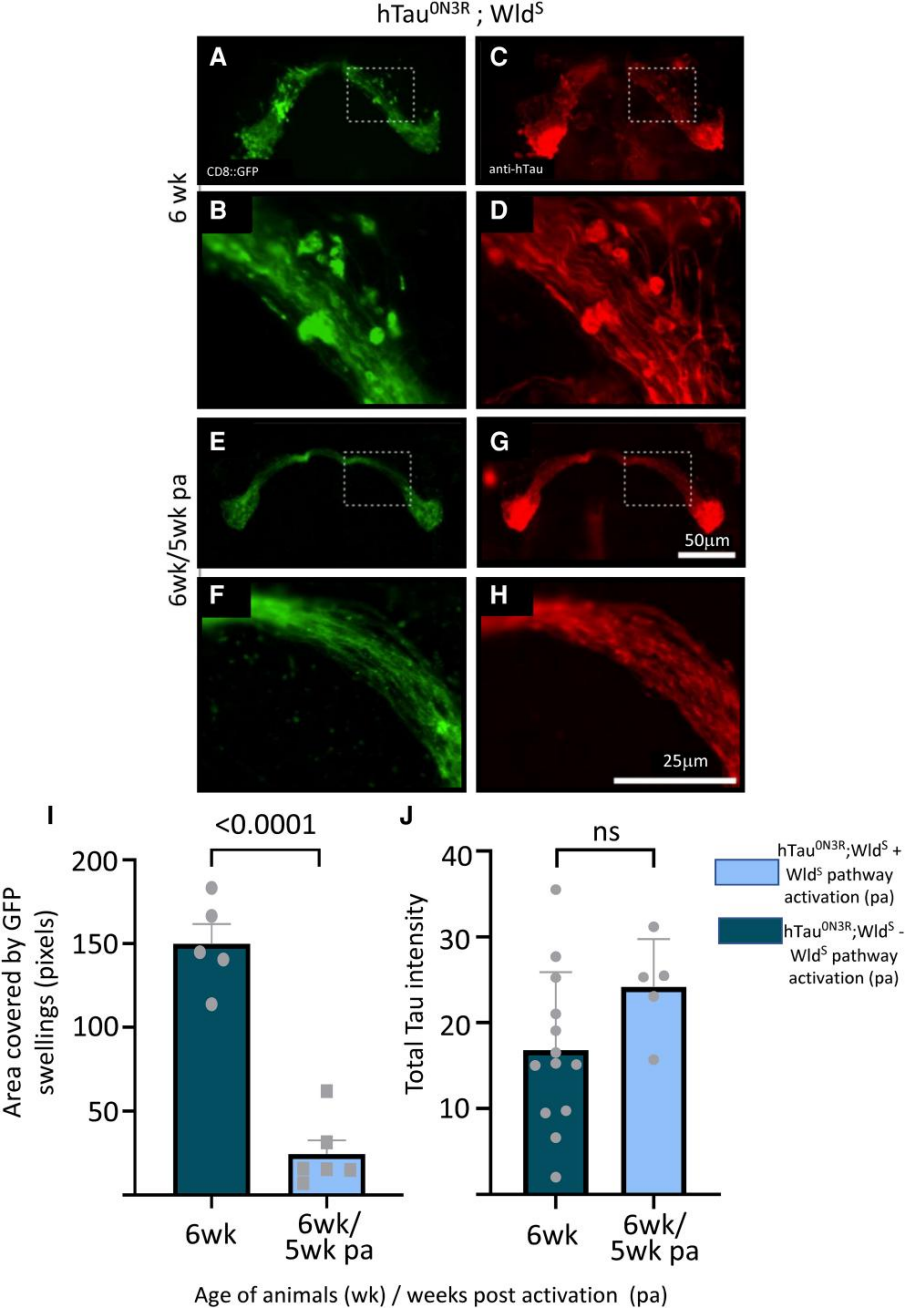
**Tau expression in naïve hTau^0N3R^;Wld^S^-expressing axons is not different to hTau^0N3R^;Wld^S^ axons where Wld^S^ pathway has been activated.** Immunostaining of the membrane-bound CD8-GFP protein shows extensive membrane fragmentation indicative of axonal degeneration in naïve hTau^0N3R^;Wld^S^ neurons at 6 weeks (**A** and **B**). Human tau is found within both the axonal processes as well as axonal swellings as visualized by a polyclonal anti-tau antibody (**C** and **D**). No such membrane fragmentation is evident in 6-week-old hTau^0N3R^;Wld^S^-expressing axons even 5 weeks after Wld^S^ pathway activation (pa) (**E** and **F**) despite persisting human tau levels (**G** and **H**). (**I**) Area covered by GFP-positive swellings indicative of axonal degeneration measured in the same animals in which tau levels were examined. Despite the persistence of human tau in these hTau^0N3R^;Wld^S^ axons where the Wld^S^ pathway was activated, tau-mediated degeneration, is significantly reduced. *n* = 5–6; each data point corresponds to an animal (unpaired two tailed *t*-test). (**J**) Quantification shows no differences in levels of total human tau between 6-week naïve hTau^0N3R^;Wld^S^-expressing axons and 6-week hTau^0N3R^;Wld^S^-expressing axons where the Wld^S^ pathway activation (pa) had occurred for 5 weeks respectively. *n* = 5–13; each data point corresponds to an animal; (unpaired two tailed *t*-test) Values are presented as the mean ± SD. hTau^0N3R^, 0N3R human tau isoform; Wld^S^, slow Wallerian degeneration.

The most parsimonious explanation for this curious phenomenon of injury-activated protection against hTau^0N3R^ pathology may simply be that hTau is lost from injured hTau^0N3R^;Wld^S^ axons and therefore cannot exert its detrimental effects to cause axonal degeneration. To investigate this, hTau immunoreactivity was assessed in hTau-expressing animals with and without Wld^S^ pathway activation, and both the total amount of hTau and its cellular localization were examined. No significant differences were found in hTau distribution or total hTau expression between naive hTau^0N3R^;Wld^S^ bigenics (Fig. 5C and D) and the hTau^0N3R^;Wld^S^ bigenics that had Wld^S^ pathway activation (Fig. 5G and H). Importantly hTau staining persisted in injured hTau^0N3R^;Wld^S^ axons even 5 weeks after Wld^S^ activation (Fig. 5G and H), and there was no difference in total hTau levels (Fig. 5J). Similar findings were evident at an earlier 3-week time point (Supplementary Fig. 2). This is remarkable because it implies that despite expression within the axon for up to 6 weeks, hTau has not caused degeneration in the hTau^0N3R^;Wld^S^ axons once the Wld^S^ pathway is activated (as seen by the dramatic and significant reduction in axonal swellings in these hTau^0N3R^;Wld^S^ axons following Wld^S^ activation, Fig. 5A and B and Fig. 5E and F—quantified in Fig. 5I). This begs the question as to how activation of the Wld^S^ pathway protects against the human tau induced degeneration in the face of persistent tau levels.

**Figure 6:**
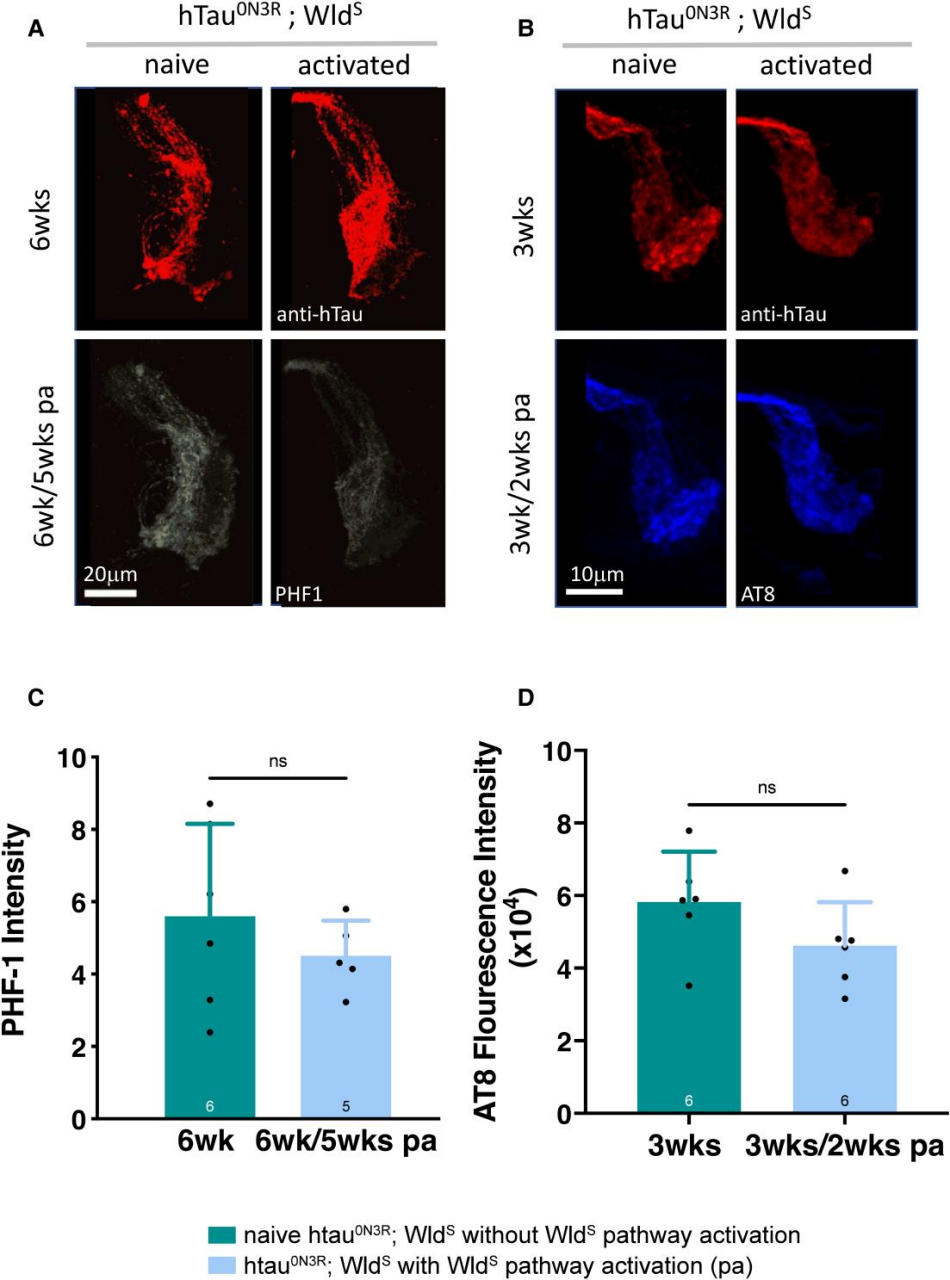
**Phosphorylated hTau levels in young and old naïve hTau^0N3R^;Wld^S^-expressing axons compared to that found in young and old hTau^0N3R^;Wld^S^ axons where Wld^S^ pathway had been activated.** (**A** and **C**) Tau phosphorylation at the ser/thr 396/404 site was assessed by quantification of fluorescence intensity after immunohistochemistry with the PHF1 antibody in naïve htau^0N3R^;Wld^S^-expressing axons at 6 weeks compared to those of the same genotype that had Wld^S^ pathway activation (pa) for 5 weeks. There was no significant difference between these two groups (*n* = 5–6; each data point corresponds to an animal; *n* > 0.05 unpaired *t*-test). (**B** and **D**) Tau phosphorylation at the ser 202/404 site was assessed by quantification of fluorescence intensity after immunohistochemistry with the AT8 antibody in naïve htau^0N3R^;Wld^S^-expressing axons at 3 weeks compared to those of the same genotype that had Wld^S^ pathway activation (pa) for 2 weeks. There was no significant difference between these two groups (*n* = 6; each data point corresponds to an animal; unpaired *t*-test *P* > 0.05). Values are presented as the mean ± SD. hTau^0N3R^, 0N3R human tau isoform; Wld^S^, slow Wallerian degeneration.

Since hyper-phosphorylation has been shown to mediate tau toxicity in many *Drosophila* models,^5,7,56^ it is conceivable that the activated Wld^S^ pathway is altering the degenerative changes by reducing the levels of phosphorylated hTau. To investigate this, hTau phosphorylated at the ser396/404 site (detected by the PHF-1 antibody^57^) was quantified in 6-week hTau^0N3R^;Wld^S^ axons with and without Wld^S^ pathway activation. Though there was a trend for a reduction in hTau phosphorylated at the PHF-1 site between naïve hTau^0N3R^;Wld^S^ animals and those that had the Wld^S^ pathway activation (Fig. 6A), this was not significant (Fig. 6C). However, it is conceivable that phosphorylation at other epitopes is affected and even that the effects are evident at earlier time points after the Wld^S^ pathway activation. To investigate this, phosphorylation at another disease-relevant epitope, ser202/thr205 (detected by the AT8 antibody^58^) was probed in 3-week hTau^0N3R^;Wld^S^ axons with and without Wld^S^ pathway activation. Tau phosphorylation at this site and this earlier time point also appeared to decline in hTau^0N3R^;Wld^S^ axons that were protected by Wld^S^ pathway activation compared to naïve unprotected htau^0N3R^;Wld^S^ axons (Fig. 6B), but as with the PHF1 site, this did not reach significance (Fig. 6D).

**Figure 7:**
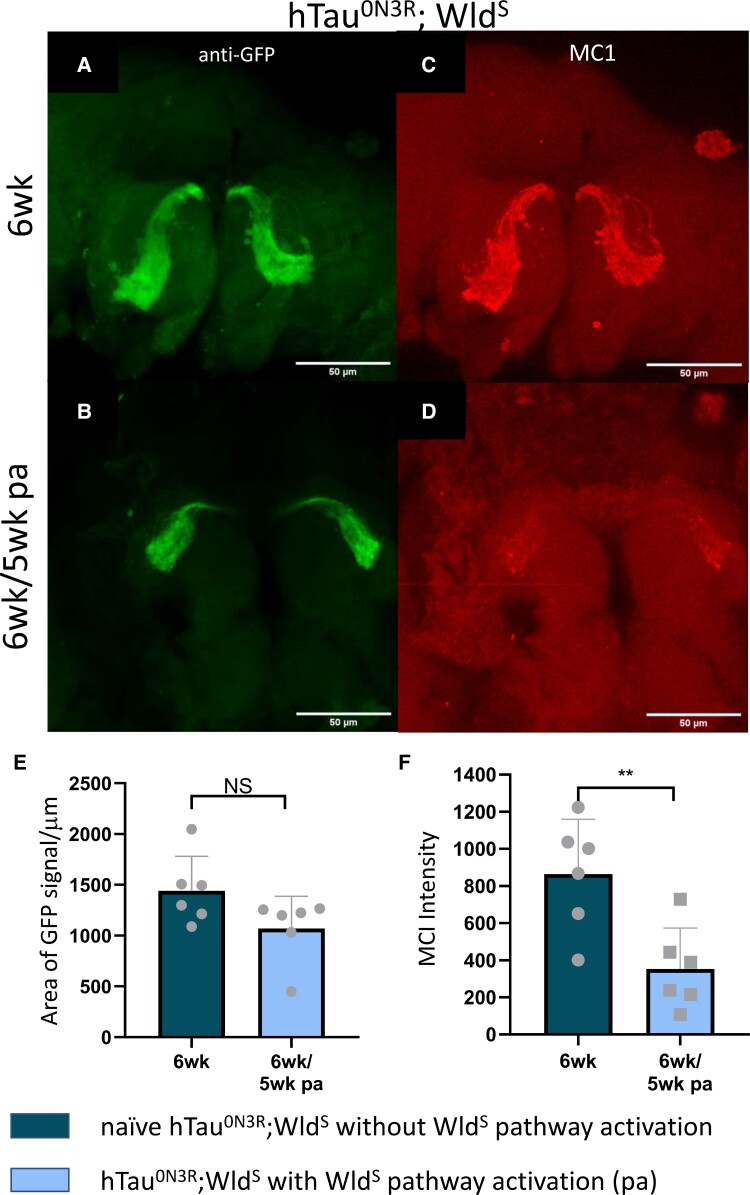
**Misfolded hTau expression in naïve hTau^0N3R^;Wld^S^-expressing axons compared to hTau^0N3R^;Wld^S^ axons where Wld^S^ pathway had been activated.** Visualization of the membrane-bound CD8-GFP protein (**A** and **B**) and misfolded MC1 positive tau (**C** and **D**) in naïve hTau^0N3R^;Wld^S^-expressing axons at 6 weeks (**A** and **C**) as well as those of the same genotype that had Wld^S^ pathway activation (pa) for 5 weeks (**B** and **D**). MC1 levels are significantly reduced in hTau^0N3R^;Wld^S^-expressing axons that have had Wld^S^ pathway activation (pa) for 5 weeks (**D** and **F**) (*n* = 6; each data point corresponds to an animal; **P = 0.0069 unpaired two-tailed *t*-test). This was not due to a non-specific loss of neurons as there was no significant difference in area occupied by membrane-bound CD8-GFP between the two groups (**E**) (*n* = 6; each data point corresponds to an animal; *P* > 0.05 unpaired two-tailed *t*-test). Values are presented as the mean ± SD. hTau^0N3R^, 0N3R human tau isoform; Wld^S^, slow Wallerian degeneration.

We next examined whether misfolding, another known pathological modification in tau,^59^ may underpin the Wld^S^ pathway activation’s protection against tau-mediated degeneration. Misfolded tau can be detected using conformationally sensitive antibodies like MC1.^59^ MC1 immunoreactivity was assessed in both 3- and 6-week naïve hTau^0N3R^;Wld^S^ axons (Supplementary Fig. 3 and Fig. 7C) or in 6-week htau^0N3R^;Wld^S^ axons that had Wld^S^ pathway activation for 5 weeks (Supplementary Fig. 3 and Fig. 7D). There was a trend for MC1 immunoreactivity to decline in the hTau^0N3R^;Wld^S^ axons that had Wld^S^ pathway activation when compared to naïve hTau^0N3R^;Wld^S^ axons at 3 weeks (Supplementary Fig. 3), and this became significant by 5 weeks (Fig. 7D and F). This reduction in MC1 immunoreactivity was not due to loss of neurons as evident by no significant change in membrane-bound CD8::GFP in the same animals (Fig. 7A and B and E).

This data collectively implies that Wld^S^ pathway activation results in a reduction of misfolded tau species, and this may contribute to the mechanism by which it confers protection against tau-mediated degeneration.

## Discussion

The axonal compartment of neurons is susceptible to tau-mediated dysfunction and degeneration making it a potential therapeutic target in the treatment of neurodegenerative disease. This study demonstrates that when the pathway downstream of Wld^S^ is activated in hTau^0N3R^;Wld^S^ axons, tau-mediated axonal swellings were prevented from forming. Significantly, in animals allowed to develop axonal swellings due to hTau^0N3R^ expression, any further progression of pathology was halted after the Wld^S^ pathway was activated. This protective effect was seen without alterations in total tau, but there was a significant reduction in misfolded tau. Understanding the mechanisms by which activation of the Wld^S^-protective pathway negates tau-mediated axonal degeneration could yield important insight into how axons degenerate in tauopathy and other similar chronic degenerative conditions and provide novel disease-modifying targets that emulate this protective effect.

### Variable impact of Wld^S^ overexpression in previous models of neurodegeneration: can this be explained by the need to activate the pathway downstream of Wld^S^ to uncover neuroprotection?

Previous studies of Wld^S^ in models of chronic neurodegeneration have indicated that expression of Wld^S^ has variable effects on disease phenotypes.^19^ This was also evident in our study where no rescue of hTau^0N3R^-mediated neuronal dysfunction or degeneration was seen in either larvae or adults stages following mere co-expression of Wld^S^. Similar results were reported in the *SOD1-G93A* model of motor neuron disease.^51^ Like this, there is a large body of conflicting evidence of Wld^S^ sensitivity in chronic neurodegenerative diseases displaying Wallerian-like degeneration. Wld^S^ did not alter axonal degeneration in models of prion disease,^50^ motor neuron disease^51,49^ and hereditary spastic paraplegia.^60^ In contrast, in models that investigate degeneration with an acute onset, such as toxic neuropathy,^39^ ischaemic injury^53^ and MPTP-induced Parkinsonism,^35,54^ a protective effect of Wld^S^ is reported.

One interpretation of the dissimilar effect of Wld^S^ in models of acute and chronic neurodegeneration and their variable sensitivity to Wld^S^ could be a different mechanism of axonal degeneration occurring in acute compared with chronic neurodegenerative conditions. Another explanation for the variable sensitivity to Wld^S^ in models of chronic neurodegeneration could simply be that its protective effect is a general delaying of degeneration that is not always apparent in the time period assayed in the chronic models in question. Our data imply that this is unlikely to be the case since no protective effect emerged at even very late time points when Wld^S^ was simply expressed with hTau^0N3R^ (Fig. 1). Instead, we propose that another explanation may be provided by a key difference between the experimental paradigms employed to study acute degeneration, which is missing in the models of chronic neurodegeneration. This is that in all acute models, injury or insult has to be simulated to create the acute neurodegenerative condition, and this may set in motion a series of events that activate the Wld^S^-protective pathway. This is never done in models of chronic neurodegeneration so it is conceivable that in those models, the protective effect of Wld^S^ is not induced due to inadequate activation of the pathway that Wld^S^ acts upon. This would limit the impact of Wld^S^ on the ensuing neurodegeneration. Where there is partial rescue of phenotype in chronic models,^19^ the Wld^S^ pathway may start to become activated as the degeneration sets in. Activation of the Wld^S^ pathway by simulating injury or established neurodegeneration is a novel concept. There is no precedence for this idea from studies published to date because no one has reported overlaying an acute injury in a chronic model. Our data indicate that Wld^S^ behaves differently in uninjured axons compared to injured ones—the mechanisms responsible for this need to be elucidated. In particular, it will be vital for future studies to identify markers associated with Wld^S^-mediated suppression of degeneration in the injured neurons wherein we hypothesize the downstream pathway is activated, e.g. higher levels of NAD^+^ and inhibition of dSARM. Though desirable, it is beyond the scope of the current work to provide this evidence (e.g. higher NAD^+^ or inactive dSARM) in our injured hTau^0N3R^;WldS bigenics that were protected when compared to naïve hTau^0N3R^;Wld^S^ bigenics that were not.

### Activation of the Wld^S^-protective pathway prevents as well as halts progression of tau-mediated degeneration

The axonal degeneration observed in hTau^0N3R^ transgenic flies was characterized by axonal swellings, which are indicative of the early stages of axonal degeneration caused by human tau. Axonal swellings are not a feature of Wallerian degeneration in the peripheral nervous system; however, they have been described following injury in the CNS.^13,61^ Axonal swellings have also been observed in models of neurodegeneration, including Alzheimer’s disease^61‐63^ and tauopathy^9,64^ leading to suggestions that Wallerian-like degeneration is occurring in neurodegenerative diseases.

Upon activation of the Wld^S^ pathway, we did not observe axonal swellings, and there was a lack of any other feature of tau-mediated degeneration in hTau^0N3R^;Wld^S^ axons. These tau-expressing axons looked as normal as the Wld^S^-expressing controls, which previous studies have shown to be both morphologically normal, as well as physiologically functional.^31^ This protective effect was evident even when the Wld^S^ pathway was activated in the hTau^0N3R^;Wld^S^ axons at a time point after tau-mediated degeneration was established. This demonstrates that once activated, the Wld^S^-protective pathway prevents emergence of tau-mediated degeneration as well as halting progression of already established degeneration.

### What is the mechanism by which activated Wld^S^ pathway protects against hTau?

Tau-mediated degeneration is dependent upon factors including total tau levels,^65^ phosphorylation at pathological sites^42,66^ and tau misfolding and subsequent aggregation.^67^ The presence of human tau within hTau^0N3R^;Wld^S^ axons, even weeks after activation of the Wld^S^ pathway, indicates that the protection seen was not due to a reduction in total tau level as a result of loss of human tau from the axon. Whether it occurs due to a reduction in tau’s phosphorylation status is unclear as though there was a clear trend for reduced phosphorylation at the two pathological sites we examined (PHF1 ser/thr396 and 40 and AT8 ser202/thr205), this did not reach significance. Instead, it is likely to be related to the significant reduction in misfolded tau, which we detected using the conformationally sensitive MC1 antibody. This effect began to emerge at early time points (Supplementary Fig. 3) but became highly significant at the late time points post Wld^S^ pathway activation (Fig. 7).

The reduction in misfolded tau can provide a potential explanation for the protective effect of Wld^S^ pathway activation against tau-mediated toxicity because several isoforms of NMNAT, one of which is a component of Wld^S^ fusion protein,^68,23^ have been shown to prevent tau-mediated aggregation *in vitro*.^44^ Furthermore, NMNAT isoforms reportedly act as chaperones for proteostasis of tau in rodent models and have been shown to promote clearance of tau oligomers and suppression of tau-induced degeneration in *Drosophila* models of tauopathy.^44‐46^ As tau aggregation has been causally linked to tau-mediated toxicity in many experimental models of tauopathy,^69^ including *Drosophila*,^67^ it is conceivable that a reduction in misfolded tau may be suggestive of reduced tau aggregation and thus reduced tau toxicity in axons that have Wld^S^ pathway activation.

Beyond misfolded tau, it may be worth considering other points of intersection between tau-mediated degeneration and the Wld^S^ pathway. A key component of the Wld^S^ pathway is the NAD^+^ salvage pathway, which has recently also been linked with Alzheimer’s disease.^70^ As alluded to above, Wld^S^ contains NMNAT1, which is the final enzyme in the NAD^+^ salvage pathway in mammals, and the biosynthetic activity of NMNAT1 is required for the full Wld^S^-protective phenotype.^71,48^ One isoform in mammals (NMNAT2) and the sole *Drosophila* homolog dNMNAT are rapidly lost upon injury, and this is associated with degeneration.^72^ Wld^S^ is believed to compensate for the loss of NMNAT in injured Wld^S^-expressing axons, thereby preventing loss of NAD^+^ and activation of the downstream pro-degenerative pathway. dSarm/Sarm1^27^ and Highwire/PHR1^41^ are endogenous mediators of axon degeneration that affect levels of NMNAT, with Axundead^31^ and Pebbled^29^ identified downstream of the loss of NMNAT. Knockout of these endogenous mediators results in axon survival after injury. We postulate that the Wld^S^-protective pathway would not be activated in uninjured hTau;Wld^S^-expressing axons, and so its protective effects would not be evident. Upon activation, the Wld^S^ pathway may confer protection against tau-mediated degeneration because its downstream components are switched on and can interact directly with pathological tau. Alternatively, protection may be conferred indirectly due to downstream neuroprotective effects of Wld^S^ pathway activation, such as enhanced mitochondrial calcium buffering,^73^ or reduced oxidative stress^74^ negating tau-mediated dysregulation in intracellular calcium and/or tau-mediated mitochondrial dysfunction^70,75^ or oxidative stress.^56^ Future studies will be required to identify the exact point at which the activated Wld^S^ pathway intersects with and therefore protects against tau-mediated degeneration. In particular, it will be vital to explore whether modulation of downstream mediators of the Wld^S^ pathway that potentially block injury-induced axon degeneration (such as NMNAT, dSarm knockdown, axed mutants or even NAD^+^ supplementation) and also suppress tau-mediated degeneration to emulate Wld^S^ pathway activation.

## Conclusion

We show that activation of the Wld^S^ pathway reliably protects against tau-mediated axonal degeneration, almost abolishing it, and this is accompanied by a reduction in misfolded tau levels. It is vital to understand how engagement of this pathway and its downstream mediators interact with tau, whether directly or indirectly, to halt tau-mediated axonal degeneration. This will yield important clues about the mechanisms underpinning tau-mediated axonal degeneration as well as enable identification of novel drug targets that can emulate the complete protection we report here, to truly halt tau-mediated degeneration in all tauopathies.

## Supplementary Material

fcad052_Supplementary_Data

## Data Availability

Data are available on the University of Southampton databases that can be accessed by contacting the corresponding author.

## Abbreviations

BSA =: bovine serum albumin
CNS =: central nervous system
GFP =: green fluorescent protein
h =: hourly
hTau^0N3R^ =: human tau protein (0N3R isoform)
MPTP =: 1-methyl-4-phenyl-1,2,3,6-tetrahydropyridine
NAD =: nicotinamide adenine dinucleotide
NMNAT =: nicotinamide mononucleotide adenylyl transferase
ORN =: olfactory receptor neuron
OR47b =: olfactory receptor neuron 47b
pa =: pathway activation
SARM =: sterile-alpha and TIR motif containing 1
SOD =: superoxide dismutase
Wld^S^ =: slow Wallerian degeneration
