# Supplementary material for: Tau-mediated axonal degeneration is prevented by activation of the WldS pathway
Source: Brain Commun. 2023 Mar 9;5(2):fcad052. doi: 10.1093/braincomms/fcad052 (PMC10066515; doi:10.1093/braincomms/fcad052)
Supplement: fcad052_Supplementary_Data [file fcad052_supplementary_data.pdf]

Stubbs et al Supplementary Figures

Supplementary Figure 1: Axotomy paradigm activates the pathway downstream of Wld^S as evidenced by suppression of axonal degeneration

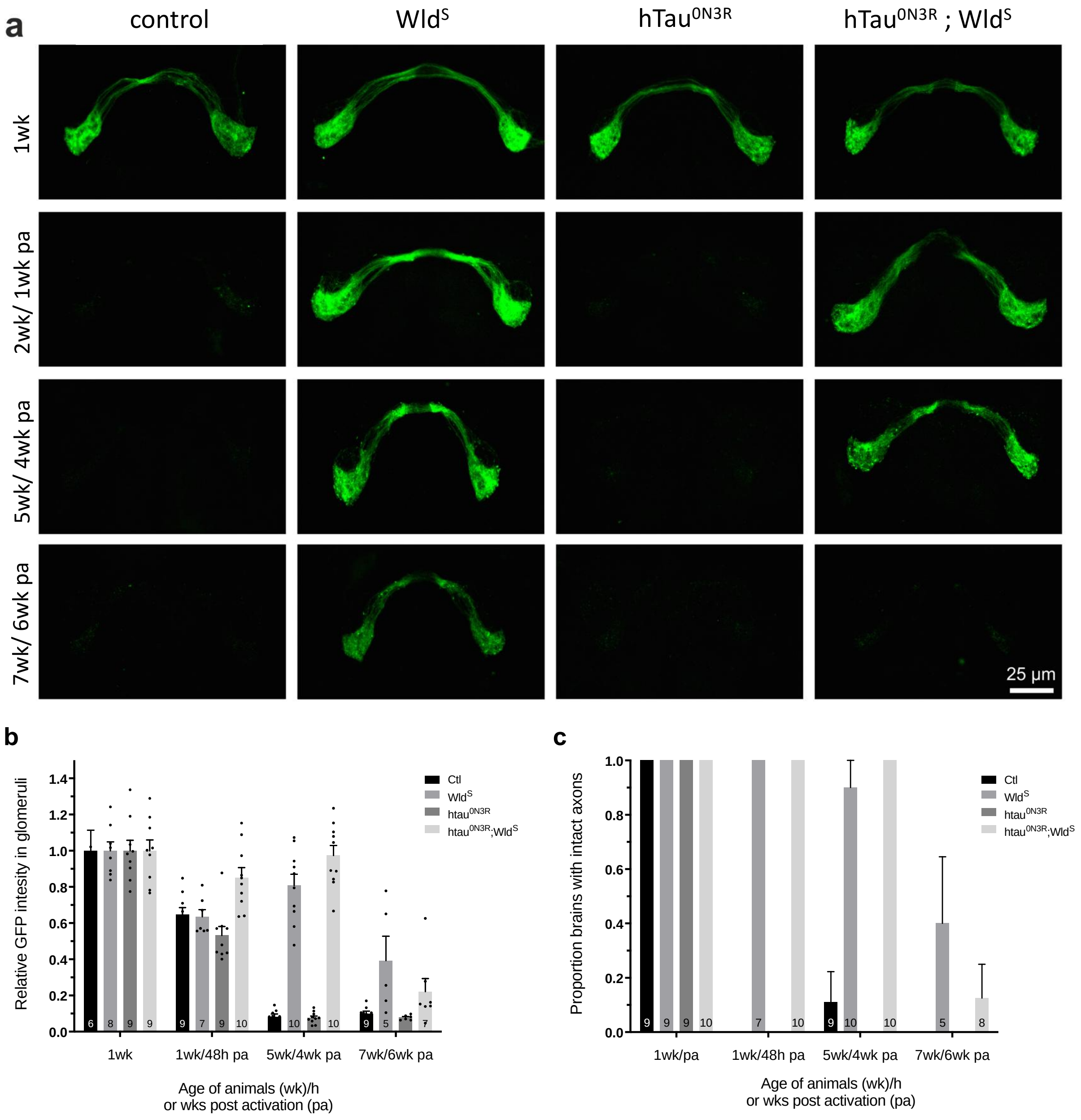

ORNs were axotomised to activate the pathway downstream of Wld^S at 1wk post ecclosion, and brains were dissected and imaged at the indicated time points post activation (pa). At 2wk post ecclosion /1wk pa, control (ctl) and hTau^{0N3R} axons have degenerated, but Wld^S and hTau^{0N3R};Wld^S brains are intact. b) Measuring GFP intensity in the antennal lobe glomeruli indicates a robust delay in axonal degeneration in Wld^S brains expressing animals. c) Scoring of intact axons reveals the majority of axons expressing Wld^S, whether alone or with hTau are intact up to 5wks post ecclosion/4wk pa, but degenerate after this point. Values are presented as the mean ± SEM. n=8-14 each data point corresponds to an animal. *P<0.05, **P<0.01, ***P<0.001. (ANOVA with Bonferroni's multiple comparisons).

<u>Supplementary Figure 2: Wld^S pathway activation does not lead to significant changes in tau levels at early time points</u>

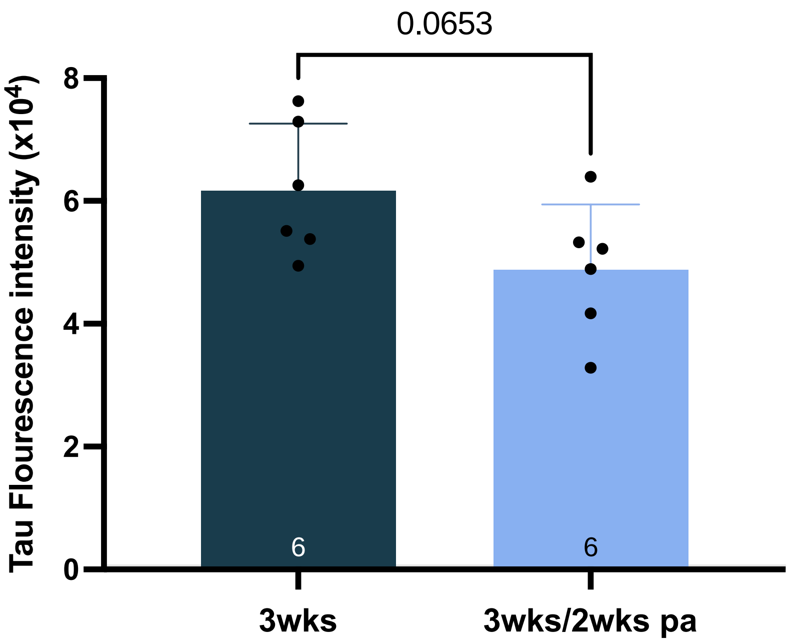

Naïve hTau^{0N3R};Wld^S without Wld^S pathway activation

hTau^{0N3R};Wld^S with Wld^S pathway activation (pa)

Human tau levels in 3wk hTau^{0N3R};Wld^S ORNs 2wks after Wld^S pathway activation are not significantly different from those found in naïve 3wk hTau^{0N3R};Wld^S ORNs that have not had Wld^S pathway activation. (n=6; each data point corresponds to an animal; p=0.07 unpaired two-tailed t test).

Supplementary Figure 3: There is a trend for misfolded tau to decrease following Wld^{S} pathway activation at early time points

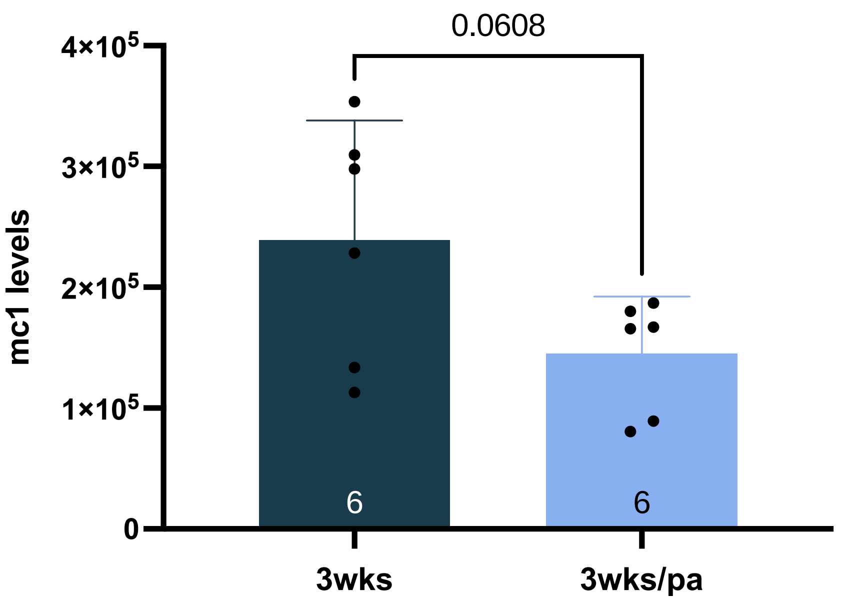

Naïve hTau^{0N3R};Wld^{S} without Wld^{S} pathway activation

hTau^{0N3R};Wld^{S} with Wld^{S} pathway activation (pa)

Misfolded tau levels (probed using the MC1 antibody) in 3wk hTau^{0N3R};Wld^{S} ORNs 2wks after Wld^{S} pathway activation appeared to decline compared to those found in naïve 3wk htau^{0N3R};Wld^{S} ORNs that have not had Wld^{S} pathway activation. However, this trend was not significant (n=6; each data point corresponds to an animal; p=0.06 unpaired two-tailed t test).
